# The nonlinear association between apolipoprotein B to apolipoprotein A1 ratio and type 2 diabetes: Erratum

**DOI:** 10.1097/MD.0000000000006821

**Published:** 2017-04-28

In the article “The nonlinear association between apolipoprotein B to apolipoprotein A1 ratio and type 2 diabetes”,^[1]^ which appeared in Volume 96, Issue 1, the authors’ affiliations appeared incorrectly. Dr. Yong Mao is affiliated with the Department of Epidemiology and Health Statistics, School of Public Health, Kunming Medical University, Kunming. Dr. Yang Xu is affiliated with the Department of Anesthesiology, Xiangyang No. 1 People's Hospital, Hubei University of Medicine, Xiangyang. Dr. Leihong Lu is affiliated with the Department of Dermatology, Linyi People's Hospital, Linyi, China.
